# Rectal Anaesthesia

**Published:** 1907-11

**Authors:** 


### RECTAL ANAESTHESIA.

Cunningham describes his method as follows: To obtain the best results it is essential that the bowels should be thoroughly cleaned out. It has been his custom to give two ounces of a saturated solution of magnesium sulphate on the evening before operation, and early the following morning a large suds enema. Just before going to the operating table another similar enema is given. The ether breakfast has consisted of two ounces of beef tea. The apparatus which he has used on all his later cases and which has been the most satisfactory, consists of a bottle, the body of which is seven and a half inches in height, five inches being used for ether space, two and a half inches and the neck for vapor space. The diameter is four inches and the capacity on the ether space twenty-nine ounces, so that a large amount of ether may be used without materially lowering the ether column. The afferent tube which leads to the bottom of the ether column ends in a bulb with several small perforations so that the air ascends in several small bubbles. The stopper and the connections should be tight. The bottle is placed in a water bath at a temperature of between 80 degrees and 90 degrees F. Ether boils at 98.6 degrees F. It is desirable to keep the temperature below this point. By keeping the ether as warm as possible without boiling, the air forced in by the bulb is more easily saturated. If the operation is a long one it may be desirable to renew the temperature of the bath. The efferent tube should be sufficiently long to allow moving the wash bottle away in case the operator wishes to change his position from one side of the table to the other. The afferent tube should be of sufficient length to allow the etherizer to inspect the patient from head to foot still retaining the bulb in hand. After narcosis is complete two or three squeezes of the bulb a minute will usually suffice to keep it so. It is noteworthy that the patients may be "run light," as they usually respond rapidly to the injections after being once etherized. If the patient becomes too profoundly anaesthetized the efferent tube should be disconnected and such ether gas as is in the bowel forced out through the rectal tube by abdominal massage. An oxygen tank should be connected with the rectal tube and this gas made to distend the bowel. Artificial respiration and stimulation should be resorted to in the usual manner. When the operation is complete it has been his custom to expel as much of the gas ether as possible by massage of the abdomen with the rectal tube still in position. Briefly summarized, the points of interest are as follows: There is no comparatively little ether used. There is no stage of excitement. Vomiting seldom occurs. Bronchial secretions are absent. There is a comparatively quick ether recovery. The bowels are slightly constipated. Unless the bowel is free from faeces it is difficult to produce narcosis.—Boston Med. & Surg. Journal. Sept. 12th, 1907.

